# Applying the Health Belief Model to Characterize Racial/Ethnic Differences in Digital Conversations Related to Depression Pre- and Mid-COVID-19: Descriptive Analysis

**DOI:** 10.2196/33637

**Published:** 2022-06-20

**Authors:** Ruby Castilla-Puentes, Jacqueline Pesa, Caroline Brethenoux, Patrick Furey, Liliana Gil Valletta, Tatiana Falcone

**Affiliations:** 1 Janssen Research & Development, LLC Titusville, NJ United States; 2 Center for Public Health Practice Drexel University Philadelphia, PA United States; 3 Hispanic Organization for Leadership and Advancement Johnson & Johnson, Employee Resource Group New Brunswick, NJ United States; 4 Janssen Scientific Affairs, LLC Titusville, NJ United States; 5 CulturIntel, Inc New York, NY United States; 6 Department of Psychiatry and Psychology Cleveland Clinic Lerner College of Medicine Cleveland, OH United States

**Keywords:** depression, COVID-19, treatment, race/ethnicity, digital conversations, health belief model, artificial intelligence, natural language processing

## Abstract

**Background:**

The prevalence of depression in the United States is >3 times higher mid-COVID-19 versus prepandemic. Racial/ethnic differences in mindsets around depression and the potential impact of the COVID-19 pandemic are not well characterized.

**Objective:**

This study aims to describe attitudes, mindsets, key drivers, and barriers related to depression pre- and mid-COVID-19 by race/ethnicity using digital conversations about depression mapped to health belief model (HBM) concepts.

**Methods:**

Advanced search, data extraction, and artificial intelligence–powered tools were used to harvest, mine, and structure open-source digital conversations of US adults who engaged in conversations about depression pre- (February 1, 2019-February 29, 2020) and mid-COVID-19 pandemic (March 1, 2020-November 1, 2020) across the internet. Natural language processing, text analytics, and social data mining were used to categorize conversations that included a self-identifier into racial/ethnic groups. Conversations were mapped to HBM concepts (ie, perceived susceptibility, perceived severity, perceived benefits, perceived barriers, cues to action, and self-efficacy). Results are descriptive in nature.

**Results:**

Of 2.9 and 1.3 million relevant digital conversations pre- and mid-COVID-19, race/ethnicity was determined among 1.8 million (62.2%) and 979,000 (75.3%) conversations, respectively. Pre-COVID-19, 1.3 million (72.1%) conversations about depression were analyzed among non-Hispanic Whites (NHW), 227,200 (12.6%) among Black Americans (BA), 189,200 (10.5%) among Hispanics, and 86,800 (4.8%) among Asian Americans (AS). Mid-COVID-19, a total of 736,100 (75.2%) conversations about depression were analyzed among NHW, 131,800 (13.5%) among BA, 78,300 (8.0%) among Hispanics, and 32,800 (3.3%) among AS. Conversations among all racial/ethnic groups had a negative tone, which increased pre- to mid-COVID-19; finding support from others was seen as a benefit among most groups. Hispanics had the highest rate of any racial/ethnic group of conversations showing an avoiding mindset toward their depression. Conversations related to external barriers to seeking treatment (eg, stigma, lack of support, and lack of resources) were generally more prevalent among Hispanics, BA, and AS than among NHW. Being able to benefit others and building a support system were key drivers to seeking help or treatment for all racial/ethnic groups.

**Conclusions:**

There were considerable racial/ethnic differences in drivers and barriers to seeking help and treatment for depression pre- and mid-COVID-19. As expected, COVID-19 has made conversations about depression more negative and with frequent discussions of barriers to seeking care. Applying concepts of the HBM to data on digital conversation about depression allowed organization of the most frequent themes by race/ethnicity. Individuals of all groups came online to discuss their depression. These data highlight opportunities for culturally competent and targeted approaches to addressing areas amenable to change that might impact the ability of people to ask for or receive mental health help, such as the constructs that comprise the HBM.

## Introduction

Worldwide, depression is the leading cause of years lost due to disability and is associated with excess mortality [1]. Data from the 2019 National Health Interview Survey showed that 18.5% of US adults had symptoms of depression, which can include depressed mood, lack of interest or pleasure in daily activities, weight loss, sleep disturbances, psychomotor issues, lack of energy, feelings of worthlessness or guilt, difficulty concentrating, and suicidal thoughts or actions, in the preceding 2 weeks [2,3].

The same survey data indicated that adult non-Hispanic Whites (NHW) and non-Hispanic Blacks were the most likely to have experienced depression symptoms in the preceding 2 weeks (19.3% each), followed by Hispanic adults (16.9%) and Asian adults (10.2%) [2]. Furthermore, data suggest that the prevalence of depression symptoms in the United States was more than threefold higher during the COVID-19 pandemic than before the pandemic [4]. Despite the significant prevalence of depression symptoms across racial/ethnic groups, the likelihood of receiving treatment for depression is significantly lower for Black Americans (BA), Hispanics, and Asian Americans (AS) compared with NHW [5,6]. Prior to the COVID-19 pandemic, barriers existed that prevented these ethnic groups from seeking mental health care, which contributed to the lower likelihood of receiving treatment. Such barriers include an increased stigma with regard to experiencing depressive symptoms, a lack of education/health literacy, cultural and language barriers, not being able to access a health care professional (HCP) because of the inability to leave or miss work, or prior experience with mistreatment/misdiagnosis by an HCP [5,7-17]. Additionally, individuals in these groups may be fearful of seeking treatment for their depression if they believe they will be met with racism or threats of deportation [12,13,18]. As the pandemic continues, the specific effects of COVID-19 on drivers and previously existing barriers to seeking help or treatment for depression are not well understood.

The health belief model (HBM) is a theoretical framework that can be used to better understand help- and treatment-seeking behaviors and has previously been used among those with depression [19,20]. The HBM considers the value one assigns to maintaining wellness or seeking treatment in the face of an illness and one’s beliefs about the effect of taking action [21]. It posits that behavior can be understood when the value an individual places on a particular outcome is known, as well as the likelihood (ie, expectation) that the action would result in the desired outcome [21].

Despite recent research efforts, there is a lack of information on how people of different racial/ethnic groups perceive and seek help or treatment for depression and how this has been impacted by the COVID-19 pandemic. Information gathered from conversations that take place online can provide new insights into how people are impacted by their disease, how to target interventions to decrease barriers to care, and how to help patients and families be more comfortable seeking and receiving mental health care.

This study applied big data and artificial intelligence (AI) techniques to analyze open-source digital conversations pre- and mid-COVID-19 by race/ethnicity to identify differences in attitudes, mindsets, key drivers, and barriers to depression care. Efforts were made to map the resulting conversations to HBM concepts in order to guide targeted outreach to specific racial/ethnic groups and inform culturally competent interventions to address depression in these communities. Such efforts have the potential to narrow gaps in known disparities with respect to mental health care.

## Methods

### Data Source

CulturIntel harvested, mined, and structured open-source digital conversations in both English and Spanish from adults who engaged in conversation about depression pre- (February 1, 2019-February 29, 2020) and mid-COVID-19 (March 1, 2020-November 1, 2020).

Only digital conversations originating from US internet protocol addresses were analyzed. Sources of conversations included message boards (ie, any online discussion site where people can hold conversations in the form of posted messages), topical sites (ie, any site that relates to a specific topic, in this case mental health and depression), social networks (eg, Facebook, Twitter, and Instagram), content-sharing sites (eg, YouTube), blogs (ie, any regularly updated website or web page, typically 1 run by an individual or a small group), and comments (ie, any mention related to mental health or depression posted publicly in an open comment box).

Rather than using keywords, conversations were mined by the topic of depression. Discussions were identified and included if they were related to depression in general (defined by the use of the term “depression” and its adjacencies, such as “feeling depressed”), seeking help for depression (defined by the use of terms such as “help,” “looking for,” “support,” and “assistance”), and depression and COVID-19 (defined by including “depression” and “COVID-19” terms). Each unique comment across discussions/posts/sites was counted for this analysis. A single comment, if appearing repeatedly through sharing or linking, was counted and analyzed once.

### Conversant Demographics and Characteristics

After the completion of comprehensive data collection, topical and tags data were extracted using the CulturIntel methodology, with the origin and user criteria based on self-identification, and a large, unstructured “big” data set was created. Demographic characteristics were determined by scanning user profiles for self-entered data and by scanning within the text for self-identification. Demographic characteristics and comorbidities related to conversations were captured in order to better profile the population behind the conversations. To categorize conversations by racial/ethnic group, the conversations had to include a self-identifier: NHW, BA, Hispanic, or AS. The clustering and tagging of the conversations as pertaining to a specific group either defined by demographic characteristics or comorbidities was based on self-identification (ie, how people self-identify in the conversation itself or on their public profile).

Most national government agencies, such as the US Census Bureau, and many research organizations use the descriptors “Hispanics,” “Latinos,” and “Latinx” interchangeably as umbrella terms for Hispanic Americans. It is possible that members of this population may elect to self-identify as 1 or many of these identities concurrently. In this study, the descriptor “Hispanic(s)” was used to describe the ethnicity of individuals who identify as any of the above-mentioned racial/ethnic groups. Similarly, the descriptor “BA” was used to describe the ethnicity of individuals who identify as “African” or “African American,” “NHW” for those who identify as “White” without any mention of other race or ethnicity, “Caucasian,” or “of European descent,” and “AS” to describe ethnicities of Asian Pacific descent.

Where possible, conversations among those with COVID-19 or those with a loved one with COVID-19 were identified using the same self-identification methodology.

### Thematic Analysis and HBM Mapping

Natural language processing (NLP) is a subfield of AI that helps computers to process and analyze large amounts of natural human language data and is broadly considered to be the study and development of computer systems that can interpret speech and text as humans naturally speak and type it. Text analytics refers to the computer-based processes used for deriving high-quality information from text. Machine learning for NLP and text analytics involves a set of statistical algorithms and rules for identifying and recognizing parts of speech, named entities, sentiment, themes, and other aspects of text. Low-level text functions are the first processes through which any text is initially analyzed, and can include tokenization (breaking text into data, such as words or groups of words), part-of-speech tagging (identifying nouns, adverbs, adjectives of each token), named entity recognition (pretagged entities), sentiment analysis (whether data is positive, negative, or neutral and devising weighted sentiment scores), and sentence boundaries and syntax analysis [22-24]. Midlevel text functions involve extracting the real content of text, including entities, themes, topics, summaries, and intentions. Finally, a high-level text function is the application of sentiment to the text. This study used a linear support vector machines model with a classification accuracy of 90.6% [25]. Using machine learning and predefined rules, text data were tagged or annotated with examples of what the machine should look for and how it should interpret that aspect. These data were used as a training set for a statistical model, which was then given untagged text to analyze. In this study, these analyses were human assisted and included repeated training, testing, and reviewing of the program output by the CulturIntel team and the authors.

These methods were used in conjunction with social media data mining to examine patterns in data and perform a thematic analysis [23,24,26]. For the thematic analysis, CulturIntel tagged and sorted data; determined key sentiments toward depression, drivers of those sentiments, motivations and barriers to seeking help, and overarching mindsets; and assigned underlying drivers and barriers, when possible, throughout decision journey stages. Conversations in English and Spanish were analyzed together.

Components of conversations were mapped to HBM concepts to explore and understand attitudes and mindsets toward depression and key drivers and barriers to seeking help and treatment among different racial/ethnic groups (Table 1). The HBM is composed of 6 constructs that predict an individual’s readiness to enact change [21]. Perceived susceptibility describes an individual’s belief that they are at risk for the health problem or associated negative outcome. Perceived severity relates to an individual’s feelings on the seriousness of contracting an illness or disease (or leaving the illness or disease untreated). Perceived benefits refer to an individual’s perception of the effectiveness of various actions available to reduce the threat of illness or disease (or to cure illness or disease). Perceived barriers are an individual’s feelings on the obstacles to performing a recommended health action; the person weighs the effectiveness of the actions against the perceptions that it may be expensive, dangerous (eg, side effects), unpleasant (eg, painful), time-consuming, or inconvenient. Cues to action refer to the stimuli (internal or external) necessary to trigger the decision to engage in a behavior. Finally, self-efficacy is the level of an individual’s confidence in their ability to successfully perform a behavior.

Through NLP, the tone of the sentiment (ie, negative, neutral, or positive) of the conversations about depression was categorized and used as a proxy for individuals’ attitudes toward seeking help or treatment. A negative sentiment toward depression was mapped to the HBM construct of perceived susceptibility. Perceived severity was mapped to drivers of negative sentiments toward the self, the future, and the world (ie, losing the quality of life, being unable to function, feeling like a burden, feeling hopeless, dealing with uncertainty, feeling stigmatized, or feeling a lack of support). Drivers of a negative sentiment toward seeking help were mapped to the HBM construct of perceived barriers; these related to the future and the world (ie, feeling helpless, feeling stigmatized, lacking resources, facing barriers, lacking support, or dealing with misinformation). Drivers of a positive sentiment toward depression related to the self, the future, and the world (ie, improving the quality of life, making progress, having a sense of agency, finding support, or getting access to treatment) were mapped to the HBM construct of perceived benefit. A neutral sentiment toward depression (ie, seeking support and treatment, understanding their situation, or looking for information and resources) was mapped to the HBM construct of cues to action; positive drivers for seeking help related to the future and the world (ie, benefiting others, building a strong support system, being encouraged by an HCP, having access to effective help, or getting helpful knowledge) were also mapped to the cues to action construct.

A mindset is a set of beliefs that orients the way a person handles their depression. Through NLP, conversations about depression reflecting individuals’ mindsets toward treatment were classified as 1 of 4 possible mindsets. The denial and troubled mindsets make up the avoiding approach, and the overcome and empowered mindsets make up the confrontational approach. The denial mindset involves ignoring the condition and declining to actively seek treatment in order to tolerate it (eg, struggling with depression but putting it aside in order to continue to care for the family). The troubled mindset involves acknowledgement of the condition but uncertainty with regard to the next steps to take or a feeling of hopelessness with regard to improvement (eg, feeling depressed, and doubtful it will get better). The overcome mindset involves reinforcing a positive outlook (eg, after being diagnosed, realizing I will get better). The empowered mindset involves recognizing that depression is an illness and taking action to feel better (eg, although depression is challenging, feeling it is within my power to cope with my symptoms and improve my quality of life). The denial and troubled mindsets were mapped to the HBM concept of perceived barriers, and the empowered mindset was mapped to the HBM concept of self-efficacy.

**Table 1:** Mapping conversations to HBM^a^ concepts.

HBM concept	Sentiment analysis
Perceived susceptibility	Negative sentiment levels toward depression
Perceived severity	Drivers of a negative sentiment toward depression
Perceived benefits	Drivers of a positive sentiment toward depression
Perceived barriers	Denial and troubled mindsets along the path to treatment Drivers of a negative sentiment toward seeking help for depression
Cues to action	Neutral sentiment level toward depression Drivers of seeking help for depression
Self-efficacy	Empowered mindset along the path to treatment

^a^HBM: health belief model.

### Numerical Analyses

Characteristics of the overall population and key segments were described by the number of conversations among subgroups of interest. Results are descriptive in nature, and no formal statistical analyses were performed.

### Ethics Consideration

All the information gathered from the different online, open sources (topical sites [eg, Depression and Bipolar Support Alliance], message boards [eg, Beyond Blue], social networks [eg, Facebook], and blogs) is in the public domain and is deidentified. The study was exempt from Institutional Review Board approval as it used publicly available, deidentified information.

## Results

### Digital Conversations About Depression

**Figure 1:**
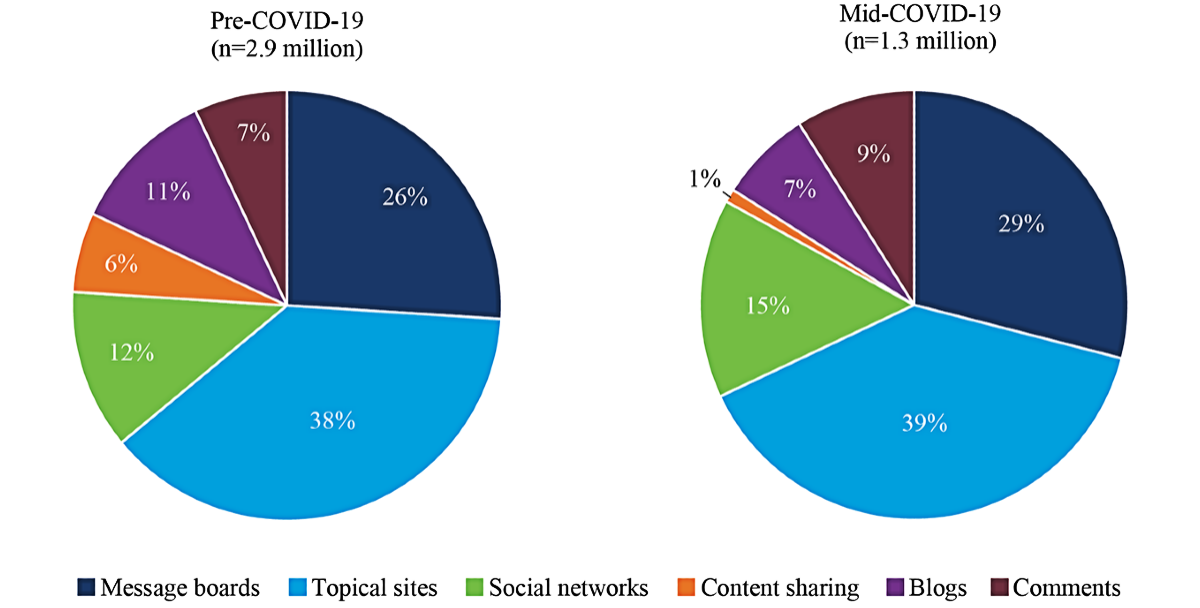
Sources of relevant digital conversations about depression pre- and mid-COVID-19.

A total of 4.2 million relevant digital conversations about depression occurring between February 2019 and November 2020 were analyzed: 2.9 million (69.1%) in the 12-month pre-COVID-19 period (February 1, 2019-February 29, 2020) and 1.3 million (30.9%) in the mid-COVID-19 period (March 1, 2020-November 1, 2020). The majority of conversations both pre- and mid-COVID-19 took place on topical sites (n=1,102,000 conversations [38%] and n=507,000 conversations [39%], respectively) or message boards (n=754,000 conversations [26%] and n=377,000 conversations [29%], respectively; Figure 1).

**Figure 2:**
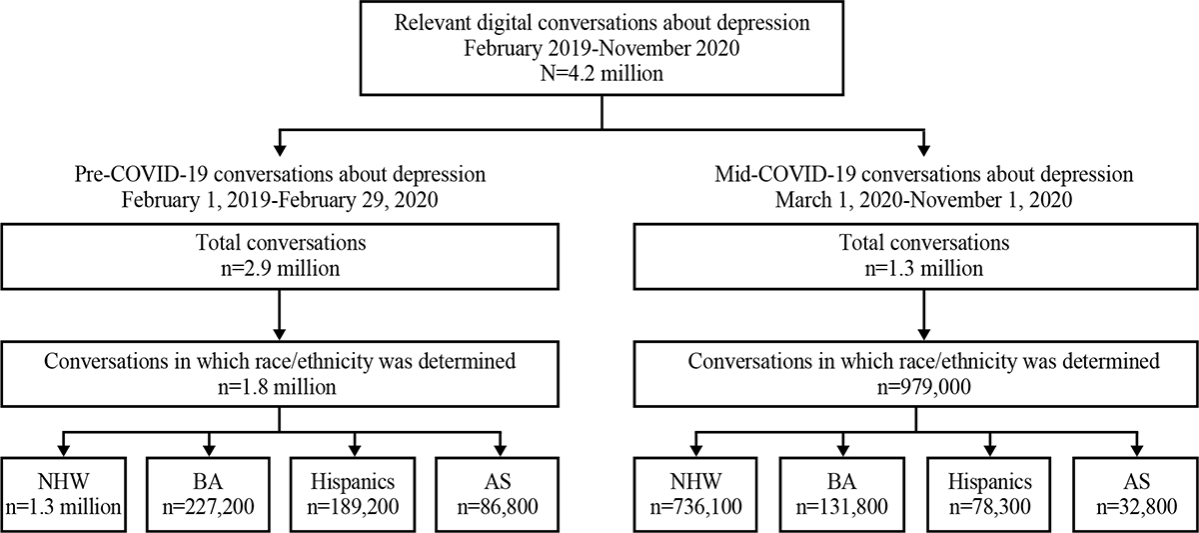
Summary of relevant digital conversations about depression pre- and mid-COVID-19. AS: Asian Americans; BA: Black Americans; NHW: non-Hispanic Whites.

Of the 2.9 million pre-COVID-19 conversations about depression, race/ethnicity was determined in 1.8 million (62.2%) conversations; 1.3 million (72.1%) conversations occurred among NHW, 227,200 (12.6%) among BA, 189,200 (10.5%) among Hispanics, and 86,800 (4.8%) among AS. Of the 1.3 million conversations mid-COVID-19, race/ethnicity was determined in 979,000 (75.3%) conversations; 736,100 (75.2%) conversations about depression occurred among NHW, 131,800 (13.5%) among BA, 78,300 (8.0%) among Hispanics, and 32,800 (3.3%) among AS (Figure 2). Mid-COVID-19, a greater proportion of conversations about depression occurred among those who mentioned having COVID-19 or having a loved one with COVID-19 among Hispanics (n=26,100 conversations, 33.3%) and BA (n=43,800 conversations, 33.2%) than AS (n=7900 conversations, 24.1%) and NHW (n=157,800 conversations, 21.4%).

### Perceived Susceptibility and Perceived Severity

**Figure 3:**
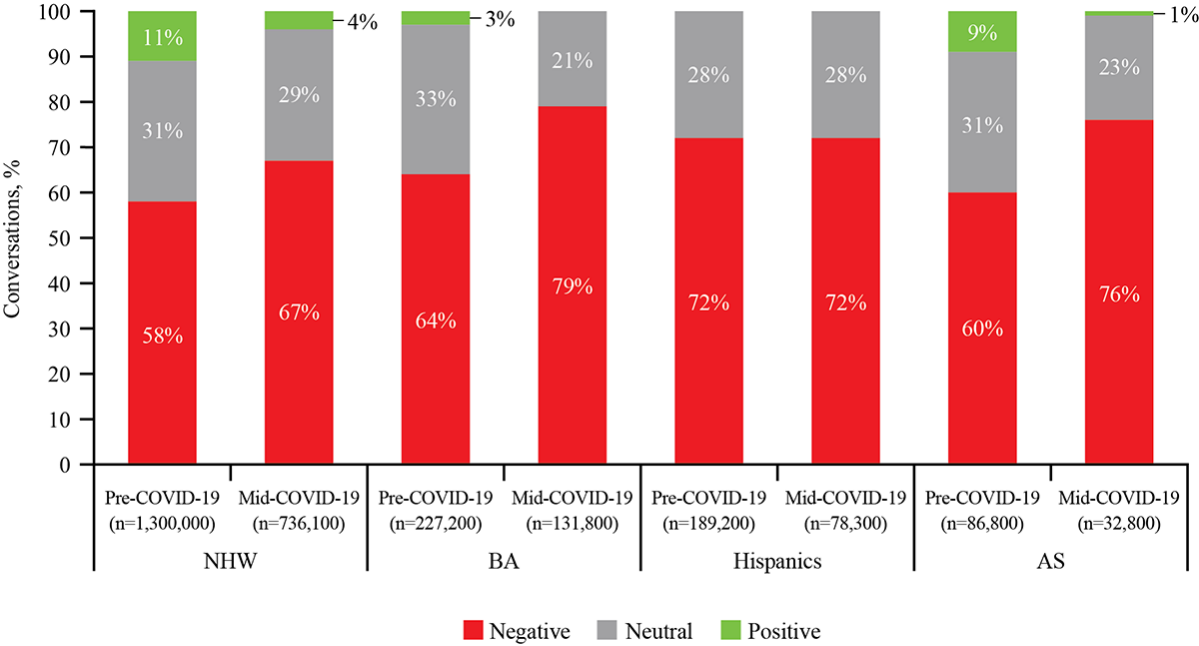
Sentiment by racial/ethnic group pre- and mid-COVID-19. AS: Asian Americans; BA: Black Americans; NHW: non-Hispanic Whites.

In the HBM, perceived susceptibility is driven by an individual’s belief that they are at risk for negative outcomes and perceived severity, which relates to beliefs about whether the problem is serious enough to warrant treatment. In this study, both perceived susceptibility and perceived severity were characterized by negative sentiment and the key themes that can be considered drivers. Across racial/ethnic groups, conversations about depression were predominantly negative and Hispanics had the highest proportion of conversations with a negative sentiment (Figure 3). The proportion of conversations that expressed a negative sentiment toward depression increased from pre- to mid-COVID-19 across most racial/ethnic groups, with the greatest increase observed among AS (52,080/86,800 [60%] to 24,928/32,800 [76%]). Mid-COVID-19, conversations among BA, Hispanics, and AS all had greater proportions of negative sentiment compared with NHW.

**Figure 4:**
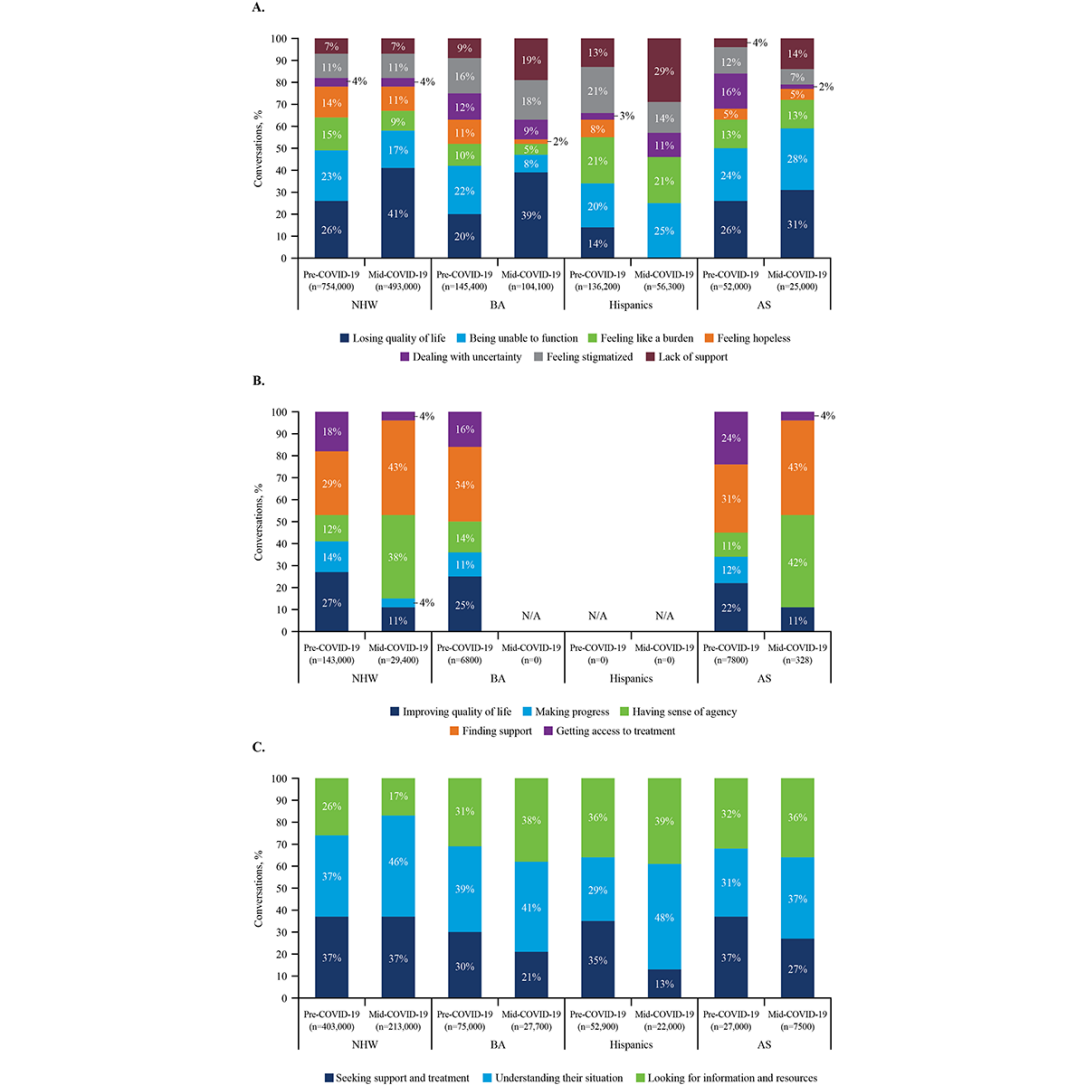
Drivers of (A) negative, (B) positive, and (C) neutral sentiment by racial/ethnic group among conversations about depression pre- and mid-COVID-19. AS: Asian Americans; BA: Black Americans; NHW: non-Hispanic Whites.

For all groups except Hispanics, losing the quality of life was the dominant topic of conversations, with substantial changes pre- to mid-COVID-19. An inability to function also emerged as a concern for all groups (Figure 4A). Feeling like a burden to others was expressed more frequently for Hispanics at both time points than for other groups. More conversations among Hispanics and BA involved stigma around depression compared with NHW and AS both pre- and mid-COVID-19. Furthermore, a higher proportion of conversations mid-COVID-19 among Hispanics, BA, and AS were related to feeling a lack of support from others (16,327/56,300 [29%], 19,779/104,100 [19%], and 3500/25,000 [14%], respectively) compared with NHW (34,510/493,000 [7%]).

### Perceived Benefits

Based on the HBM, perceived benefits relate to beliefs about the benefits of treatment; perceived benefits were mapped to drivers of a positive sentiment toward depression. Conversations revealed little positive sentiment toward depression across racial/ethnic groups. Further, a positive sentiment among NHW, BA, and AS decreased from pre- to mid-COVID-19; Hispanics displayed no positive sentiment toward depression both pre- and mid-COVID-19.

Finding support was important across the NHW, BA, and AS groups, as evidenced by the highest frequency of conversations (Figure 4B). In the NHW group pre-COVID-19, a positive sentiment toward depression was most often expressed by finding support (41,470/143,000 [29%]) and improving the quality of life (38,610/143,00 [27%]). Mid-COVID-19, finding support was discussed even more often (12,642/29,400 [43%]), and having a sense of agency seemed to become more relevant than it had been pre-COVID-19. In conversations among BA pre-COVID-19, the small amount of positive sentiment was driven by finding support (2312/6800 [34%]); a positive sentiment toward depression was absent in conversations among BA captured mid-COVID-19. Conversations in the AS subpopulation pre-COVID-19 were primarily dominated by finding support (2418/7800 [31%]) and getting access to treatment (1872/7800 [24%]). Mid-COVID-19, a positive sentiment among AS significantly decreased, but the remaining positive sentiment was tied to finding support (141/328 [43%]) and having a sense of agency (138/328 [42%]).

### Perceived Barriers

The HBM concept of perceived barriers, which is related to beliefs about internal/external barriers to treatment, was mapped to the mindset of avoiding accessing treatment for their depression and drivers of a negative sentiment toward seeking help or treatment for depression. The way a person handles their depression is related to their mindset, which can range from avoiding to confronting. Avoiding mindsets include denial (ie, ignoring the condition to tolerate it) and troubled (ie, carrying the burden without acting), while confronting mindsets include overcome (ie, reinforcing a positive mindset) and empowered (ie, taking action to feel better).

Pre-COVID-19 conversations indicate that, of all groups, Hispanics had the most avoiding mindsets (112,000/130,233, 86% of conversations), followed by BA (156,000/232,825, 67% of conversations), NHW (396,000/707,143, 53% of conversations), and AS (37,000/77,083, 48% of conversations); these mindsets were not substantially different mid-COVID-19.

All racial/ethnic groups faced external barriers to getting help for depression. Stigma was a significant driver of negative sentiment in all groups seeking help for depression both pre- and mid-COVID-19. Pre- and mid-COVID-19, BA, Hispanics, and AS all faced more external practical and logistical barriers to getting help (eg, lack of transportation/long commute to the point of care, inability to secure adequate time off or child/elderly care to engage with care) than NHW; among those seeking help for depression mid-COVID-19, 36% (5000/13,878), 31% (4000/12,903), and 29% (1000/3447) of conversations among BA, Hispanics, and AS, respectively, expressed concerns about external barriers compared with 17% (17,000/99,989) of conversations among NHW.

Of all groups, Hispanics most frequently expressed that they did not have access to a support system (mid-COVID-19, 16,327/56,300 [29%] of barriers to seeking help; Figure 4A). Hispanics also mentioned lacking resources for getting help more frequently than other racial/ethnic groups (mid-COVID-19, 23% [8000/34,781] of barriers to getting help). BA also often felt they lacked resources for getting help (mid-COVID-19, 18% [17000/94,442] of barriers to getting help).

### Cues to Action

Based on the HBM, cues to action are factors that increase treatment readiness, and these were mapped to neutral sentiments toward depression and drivers for seeking help for depression. The proportion of conversations expressing neutral sentiments toward depression pre- and mid-COVID-19 were relatively consistent among NHW and Hispanics, while conversations with a neutral sentiment decreased among BA and AS, consistent with increasingly negative conversations about depression. Conversations about understanding their situation among Hispanics increased from 15,341 (29%) of 52,900 conversations to 10,560 (48%) of 22,000 conversations pre- to mid-COVID-19 (Figure 4C).

Compared with NHW, Hispanics, BA, and AS had more conversations about looking for information and resources pre-COVID-19 (104,780/403,000 [26%], 19,044/52,900, [36%], 23,250/75,000 [31%], and 8640/27,000 [32%], respectively). Mid-COVID-19, a similar pattern was observed, with a higher proportion of conversations among Hispanics, BA, and AS related to looking for information and resources compared to NHW. There were also fewer conversations among Hispanics, BA, and AS (2860/22,000 [13%], 5817/27,700 [21%], and 2025/7500 [27%], respectively) mid-COVID-19 about seeking support and treatment compared to NHW (78,810/213,000 [37%]).

Among those seeking help for depression, being able to benefit others was a driver among all racial/ethnic groups pre-COVID-19 (less than 25% of drivers [121,000/484,000]) but was not a key driver mid-COVID-19 (less than 2% of drivers [7000/350,000]). Among Hispanics, building a strong support system and being encouraged by an HCP were key drivers to seeking help pre-COVID-19 (29% [4000/13,793] and 22% [6000/27,281] of drivers, respectively), but all positive sentiment toward depression had disappeared mid-COVID-19.

### Self-efficacy

Based on the HBM, self-efficacy, which relates to how well or poorly a person is able to cope with a given situation based on the skills they have and the circumstances they face, was mapped to having an empowered mindset about depression in this study. Of all groups, conversations among AS were most likely to indicate an empowered mindset about depression (3000/7317, 41% of conversations), followed by NHW (7000/20,588, 34%), BA (4000/13,793, 29%), and Hispanics (2000/14,285, 14%).

**Figure 5:**
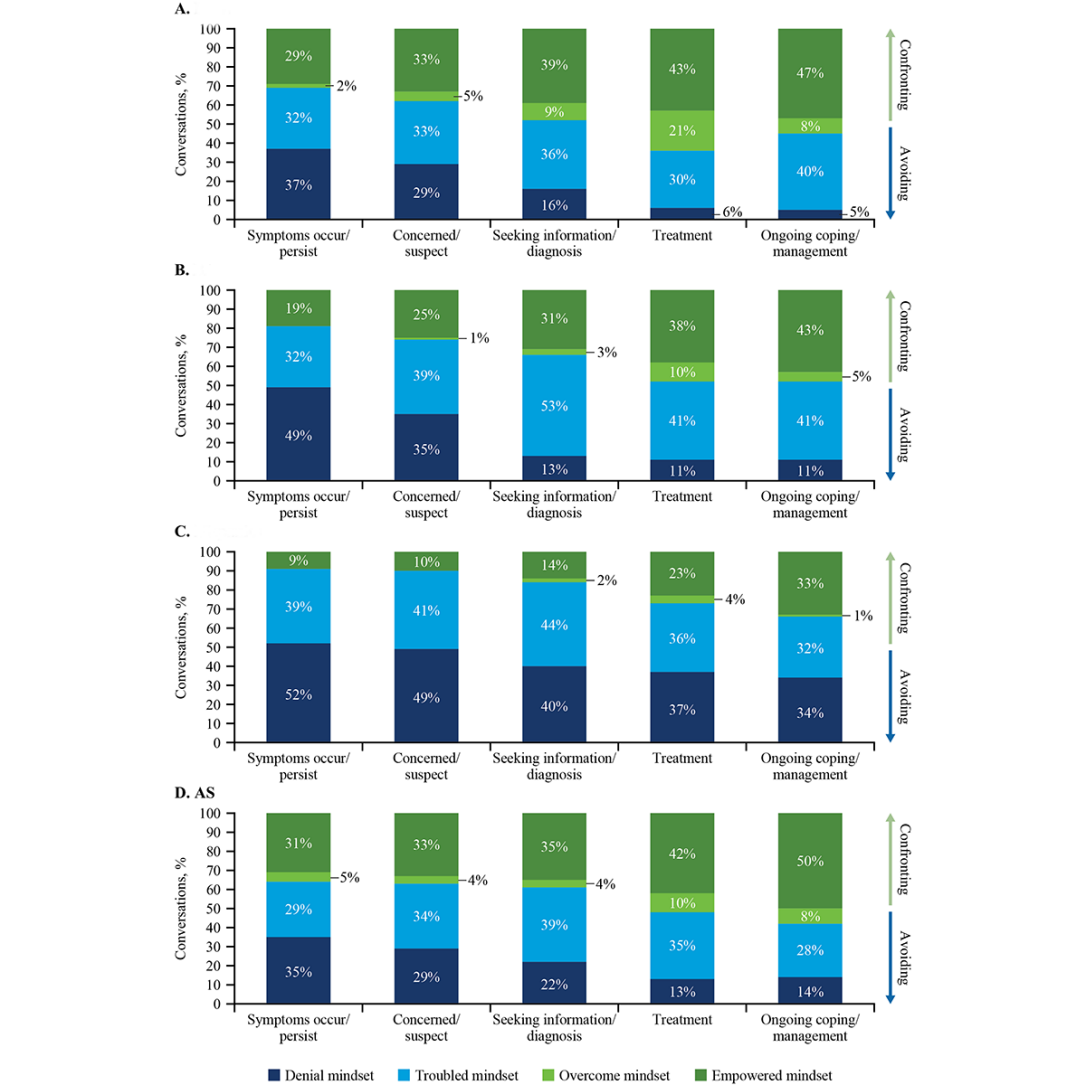
Mid-COVID-19 conversations about mindsets along the path to treatment from conversations among (A) Non-Hispanic Whites (NHW), (B) Black Americans (BA), (C) Hispanics, and (D) Asian Americans (AS).

Conversations were also examined to determine the mindsets along the path to treatment. The path to treatment starts with the occurrence or persistence of symptoms, then concern or suspicion that the individual may have depression, seeking information or a diagnosis, treatment, and finally ongoing coping and depression management. Along the path to treatment, NHW and AS displayed the highest levels of empowered mindsets (Figure 5). Conversations indicate that few Hispanics started the path to treatment with an empowered mindset (3000/33,327, 9%). Hispanics tended to display the most empowered mindset during the ongoing management stage (4000/12,125, 33%), though it was still lower proportionately than among NHW (11,000/23,404, 47%). Conversations indicate that few in the BA group started the path to treatment with an empowered mindset (4000/21,052, 19%). Across all racial/ethnic groups, the highest level of empowered mindset was observed during the ongoing management phase, suggesting that there is an opportunity to drive adherence to treatment during this phase across racial/ethnic groups.

## Discussion

### Principal Findings

This study identified online discussions about depression among individuals of all racial/ethnic groups. There were considerable differences between racial/ethnic groups in drivers and barriers to seeking help and treatment for depression pre- and mid-COVID-19. Generally, conversations about depression were more negative mid-COVID-19, with frequent discussions of barriers to seeking care.

The HBM is a value expectancy theory that has been previously used to better understand help- and treatment-seeking behaviors among those with depression [19,20]. All domains of the HBM have been associated with the likelihood that an individual is receiving treatment for depression [27]. Consistent with previous findings, this study suggests that interventions targeting different HBM concepts may have varying impacts on different ethnic groups [27].

Recently, social media data have also been applied to the COVID-19 pandemic to examine topics ranging from trends in discourse to gender differences in mental health outcomes during the pandemic [28,29], though the effects of the COVID-19 pandemic on depression among different racial/ethnic groups remained unexplored. This study further highlights the utility of online conversations for offering insight into how individuals think about and cope with their disease, including those dealing with mental health issues, such as depression [30-32]. To the best of the authors’ knowledge, this study is the first to apply HBM concepts to online conversations to provide new insights into attitudes, mindsets, and help- and treatment-seeking behaviors related to depression among different racial/ethnic groups pre- and mid-COVID-19.

In this study, the majority of conversations among all racial/ethnic groups discussing their depression online had a negative tone. Pre-COVID-19, the highest proportion of negative sentiment was observed among Hispanics, and the high rate of negative sentiment among Hispanics continued mid-COVID-19. A negative sentiment toward depression increased pre- to mid-COVID-19 in other racial/ethnic groups. Though a relatively small proportion of conversations expressed a positive sentiment toward depression, finding support from others was seen as a benefit for most groups. A previous study conducted among African Americans has shown that partnering with community members is a key factor that can facilitate engagement with health care services [33]. Therefore, efforts by community leaders and HCPs to emphasize the benefits of receiving support from one’s own community could help encourage those affected by depression to address their condition.

All racial/ethnic groups who discussed depression online had already seen some negative impacts of depression on their life. For all racial/ethnic groups, perceived barriers to treatment were both internal and external. Internal barriers were mindset driven, and the Hispanic group demonstrated the greatest unwillingness to acknowledge their depressive symptoms; these mindsets did not change with COVID-19. This aligns with previously published studies highlighting the internal stigma with regard to depressive symptoms within the Hispanic community [8,13,16,34]. External barriers to seeking treatment for depression included stigma, lack of support from others, and lack of resources. Stigma and a lack of support from others were particularly relevant for Hispanics and BA, aligning with previous studies that demonstrated how depression symptoms are perceived and responded to among members of these racial/ethnic groups [5,7-9,12,13,16,34]. To effectively help individuals overcome stigma about depression, HCPs should know how to interact with individuals with depression, including the use of person-first behavior; learn about interventions targeting unconscious biases and false beliefs; and understand how they can have an impact on their patients’ recovery [35]. Hispanics, BA, and AS were all more likely to face external barriers to treatment compared to NHW. The barriers to health care observed in this study should be considered in the context of racial/ethnic disparities in health care that have existed and continue to exist throughout the United States [5].

Being able to benefit others was a key driver to seeking help pre-COVID-19 for all racial/ethnic groups; thus, leveraging loved ones and positioning treatment as a way to help those suffering from depression offer and receive support from loved ones are key opportunities for HCPs to trigger action. Furthermore, building a strong support system was the most prominent driver to seeking help for many groups. Thus, mid-COVID-19, creating a support system while observing and promoting social distancing is critical.

Of all racial/ethnic groups, the AS group had the most empowered attitude toward depression. However, once individuals reached the ongoing management stage of the path to treatment, all groups had an increase in the proportion of conversations with an empowered mindset. This provides HCPs with an opportunity to drive adherence to treatment. The HBM can be used as a basis for developing culturally appropriate educational programs and materials that will increase adherence to treatment on an ongoing basis [33,36,37].

In summary, findings from this study demonstrated that individuals of all racial/ethnic groups come online to talk about depression. These findings also underscore the importance of considering how race/ethnicity might impact an individual’s ability to ask for or receive mental health help and the need to make resources and information available in a culturally relevant manner and in appropriate languages, where applicable.

### Limitations

Limitations of this analysis include the fact that the application of the tenets of the HBM were a priori, which may limit the replicability of these findings. Additionally, only digital conversations were considered; thus, the characteristics of those who join online communities to discuss depression may not reflect the overall population of those with depression, which may limit the reliability and generalizability of these findings. There may also be biases related to the different sources of digital conversations. The fact that only people who self-identified as a specific race/ethnicity were included in the study, that it was not possible to confirm a diagnosis of depression as patients were self-identified as having depression, and that conversations about depression were captured instead of conversations among patients with depression are also sources of possible bias. Despite these limitations, this study used advanced methodology and a large sample size to explore prevalent themes expressed by people of different racial/ethnic backgrounds and how they align with the tenets of the HBM, which can be leveraged to inform culturally competent interventions to address depression.

### Conclusion

AI-powered data analysis can contribute to better health care communications and patient engagement. This study demonstrated that there were considerable racial/ethnic differences in attitudes and mindsets toward depression and drivers and barriers to seeking help and treatment pre- and mid-COVID-19. These findings may help guide initiatives that proactively educate and empower caregivers, HCPs, and families with culturally sensitive information to contextualize and address depression in their communities. Future studies should aim to replicate these findings with psychometrically validated instruments and using additional theoretical frameworks (eg, other than the HBM) and other minority groups (eg, the lesbian, gay, bisexual, transgender, queer or questioning, intersex, and asexual [LGBTQIA+] community).

## Abbreviations

AI: artificial intelligence
AS: Asian Americans
BA: Black Americans
HBM: health belief model
HCP: health care professional
NHW: non-Hispanic Whites
NLP: natural language processing

## Data Availability

The data sets generated during or analyzed during the study are not publicly available but may be available from the corresponding author on reasonable request.
